# Race, Ethnicity, and Other Barriers to Access Dental Care During Pregnancy

**DOI:** 10.1007/s40615-024-02001-4

**Published:** 2024-04-26

**Authors:** Hyewon Lee, Richa Deshpande, Emma K. T. Benn

**Affiliations:** 1 Global Maternal and Child Oral Health Center, Seoul National University, Dental Research Institute & School of Dentistry, Seoul, South Korea; 2 Center for Scientific Diversity, Center for Biostatistics, and Department of Population Health Science and Policy, Icahn School of Medicine, New York City, USA

**Keywords:** Pregnancy, Oral health, Racial/ethnic disparities, Access to care

## Abstract

**Background:**

Historically, women of color showed poorer oral health and lower dental service utilization in the USA. These barriers to dental care during pregnancy included dental coverage, primary language, dental provider availability, safety concerns, affordability of dental care, and perceived oral health benefits during pregnancy.

**Methods:**

The purpose of this study is to examine whether race/ethnicity modified the associations between barriers to accessing dental care and dental service utilization during pregnancy. This cross-sectional study sample included 62,189 women aged 20 and older with a recent birth history in 21 states from the Pregnancy Risk Assessment Monitoring System (PRAMS) data from 2016 to 2019. We introduced a race/ethnicity by barrier interaction term to our multiple logistic regression models.

**Results:**

After adjusting for other confounders, dental insurance during pregnancy and perceived oral health benefits were associated with 4.0- and 5.6-fold higher odds, respectively, of dental service utilization during pregnancy. Statistically significant effect modification by race/ethnicity was observed in crude and adjusted analyses of the relationship between dental service utilization for all barriers included in the interaction analyses with all adjusted *p*-values < 0.001.

**Conclusion:**

The interaction analysis found that racial/ethnic disparity in visiting dentists during pregnancy was significant among women who reported these dental barriers. In contrast, such racial/ethnic disparity was substantially attenuated among women who did not report such barriers.

**Practical Implications:**

The observed racial/ethnic disparities could be mitigated by such supporting mechanisms: dental coverage, provider availability and willingness to treat pregnant women, oral health education on the safety of dental care during pregnancy, and affordable dental care costs.

**Supplementary Information:**

The online version contains supplementary material available at 10.1007/s40615-024-02001-4.

## Background

The recent Surgeon General’s report on maternal health was published with clear concern about racial and ethnic health disparities among women [1]. Non-Hispanic Black and American Indian/Alaska Native (AI/AN) women showed significantly higher rates of pregnancy-related death and morbidity than women of other racial and ethnic groups [1]. Similar findings and patterns across racial/ethnic groups of women have been observed in oral health status and dental care utilization. Historically, women of color showed poorer oral health and lower dental service utilization [2, 3]. The U.S. National Health and Nutrition Examination Survey from 1999 to 2004 showed the prevalence of untreated dental caries during pregnancy was higher among non-Hispanic Black women (45%) and Mexican American women (42%) than non-Hispanic White women (18%) [3], and a systematic review that examines a global perspective of racial-ethnic inequities in dental caries is under development [4].

In general, dental service utilization is low among pregnant women. A study based on Centers for Disease Control and Prevention (CDC) Pregnancy Risk Assessment Monitoring System (PRAMS) data reported that only about half of pregnant women had routine dental care during pregnancy [5], and the proportion of women who visited dentists during pregnancy was decreasing. Despite of the national guideline of oral health for pregnant women clearly indicating that dental care during pregnancy is safe and recommended, low dental service utilization among pregnant women persists due to lack of dental insurance, perceived ability to pay for care, difficulty finding dental providers who are willing to treat pregnant women due to liability concerns, and misconceptions about the safety of dental care during pregnancy.

Racial/ethnic disparity in dental service utilization among women is alarming. A study based on CDC PRAMS data from 2012 to 2015 showed that the proportion of non-Hispanic White women who visited dentists for cleaning during pregnancy decreased from 56.7% in 2012 to 54.4% in 2015, whereas it decreased from 42.4 to 39.8% among non-Hispanic Black women during the same time period [5]. This Black-White disparity in utilizing routine dental care during pregnancy persisted when the analysis model was adjusted by mother’s age, marital status, dental insurance, education level, previous live birth, adequacy of prenatal care, and perception of benefits of oral health care [5], which confirmed the previous findings [6]. Therefore, there is a clear role of race/ethnicity in dental care utilization among women of childbearing age.

While a previous study and its findings were significant in understanding the Black-White gap in accessing dental care during pregnancy, the study only included non-Hispanic Black and non-Hispanic White women aged 20 and older [5]. Also, a previous analysis on specific barriers to accessing dental care during pregnancy was limited as this PRAMS Phase 7 question was implemented in only five states. To fill this gap, we conducted a secondary analysis of the PRAMS Phase 8 dataset to (1) identify barriers to accessing dental care during pregnancy and (2) examine how the association between these barriers to accessing dental care is modified by race/ethnicity.

- Specific aim 1: to identify barriers to accessing dental care during pregnancy

The study examined barriers to accessing dental care during pregnancy, including dental coverage, primary language, provider availability and willing to treat pregnant women, safety concerns of dental care, affordability of dental care, and perceived oral health benefits during pregnancy. We hypothesized that women who did not have dental coverage, whose primary language is not English, who reported difficulty in finding dentists, who had safety concerns about dental care, who reported difficulty in affording dental care, or who did not perceive the benefits of oral health were less likely to visit dentists for cleaning during pregnancy. Independent variables included each barrier to accessing dental care, and the dependent variable was dental visits for cleaning during pregnancy.

- Specific aim 2: to examine how the association between these barriers to accessing dental care and dental service utilization during pregnancy is modified by race/ethnicity

We hypothesized that there would be a significant modifying effect of race/ethnicity on associations between the indicated barriers and dental visits for cleaning during pregnancy. Independent variables were all the barriers to accessing dental care (listed above in aim 1), the dependent variable was dental service utilization for cleaning during pregnancy, and the moderating variable was maternal race/ethnicity.

## Methods

### Database

PRAMS is a yearly surveillance project of the CDC and state health departments for women with a recent birth history. The detailed discussion on PRAMS collection methodology has been explained well in previous papers [5, 7]. The minimum overall response rate threshold was 55% in 2016 and 2017 and 50% in 2018 and 2019. The PRAMS consists of a survey questionnaire and birth certificate data, and the questionnaire is comprised of both core and standard questions. Core questions are asked by all states that participated in the phase, while some states include standard questions. The birth certificate variables and oral health-specific survey questions for this study are available in the Supplemental Files. In this analysis, the standard oral health question that included barriers to dental care during pregnancy was answered by 21 states, including Puerto Rico. We used SAS 9.4, RStudio version 1.4.1106, and Stata 17 to account for PRAMS’ complex weighting and oversampling methodology for the study. The data is available for analysis, and CDC issued a letter for a data access agreement. The study was exempted for the Institutional Review Board approval by Icahn School of Medicine and Mount Sinai Hospital System.

### Study Sample Description, Inclusion/Exclusion Criteria

The cross-sectional study sample included 62,189 women (for a weighted total of 3,327,782 women) aged 20 years and older with a recent birth history in 21 states that implemented the standard oral health questions about barriers to accessing dental care during pregnancy. The race/ethnicity was re-categorized as non-Hispanic White, non-Hispanic Black, Hispanic, Asian/Pacific Islanders, AI/AN, and others (Supplemental file). Maternal education was clustered in completed school years (0–11, 12, 13, or more). Health insurance status at the time of birth was assigned as private insurance, Medicaid, self-pay, and other government insurance (e.g., Tricare, Indian Health Service). Kotelchuck Index was used as an indicator for adequacy of prenatal care, which is a scoring system that considers both timings of prenatal care initiation and the number of prenatal care visits after the first visit and is categorized into an inadequate, intermediate, adequate, and adequate plus. Other variables and their operational definitions are listed in the Supplemental Files. All these variables were from the birth certificates except dental variables. Women whose responses were coded as “missing,” “don’t know,” or any other responses not listed in the variable dictionary from the analyses for race/ethnicity, age, and dental visits for cleaning were excluded.

### Overall Study Design, Strategy, Methodology, Analyses

Twenty-one states were from the PRAMS Phase 8 database (2016–2019) that implemented two sets of standard oral health questions on barriers to accessing dental care and information regarding dental care during pregnancy and met the minimum response rate for the year (Supplemental file). From these standard oral health questions, we selected potential barriers to accessing dental care based on previous literature and our own studies. These barriers were dental coverage, primary language, dental provider availability, safety concerns, affordability of dental care, and perceived oral health benefits during pregnancy (Table 1). All dental variables were self-reported, and neither dental visits nor dental insurance coverage could be confirmed from objective data. Therefore, we explained the dental coverage variable as a proxy for awareness of dental insurance. We analyzed the association between these barriers and dental service utilization (dental cleaning) during pregnancy in these 21 states.

**Table 1:** Variables from PRAMS survey

Category	• Variables	Categorization
Language variable (core questions)	• Language of the PRAMS questions	English Chinese Spanish
Dental variables (core question)	• I had my teeth cleaned by a dentist or dental hygienist during my most recent pregnancy **(dental visit for cleaning)** • I had insurance to cover dental care during my pregnancy** (dental insurance)** • I knew it was important to care for my teeth and gums during my pregnancy **(perceived oral health benefits)**	Yes No
Dental variables (standard questions in 21 states)	• I could not find a dentist or dental clinic that would take pregnant patients **(difficulty finding dentists who accept pregnant women**) • I did not think it was safe to go to the dentist during pregnancy **(safety concern to go to the dentist during pregnancy)** • I could not afford to go to the dentist or dental clinic **(difficulty affording dental care)** • I could not find a dentist or dental clinic that would take Medicaid patients **(difficulty finding dentists who accept Medicaid)**	Yes No

In order to test whether the aforementioned potential barriers (independent variables) were associated with the binary dental service utilization outcome (dependent variable), we conducted chi-square tests. To account for the potential for inflation of our type I error due to multiple testings, we applied a Benjamini and Hochberg adjustment [8]. We additionally examined the crude magnitude of the bivariate associations using logistic regression accounting for the complex survey design, and we adjusted for potential confounders subsequently. To additionally examine whether race/ethnicity modified the crude and adjusted associations between the barriers identified as statistically significant in bivariate analyses and dental service utilization, we introduced a race/ethnicity by barrier interaction term to our simple and multiple logistic regression models.

## Results

In the preliminary analysis, 62,189 individual women were included in the study. In the study population, 64.0% of women were non-Hispanic White, 11.3% non-Hispanic Blacks, 19.7% Hispanic, 1.5% Asian/pacific islanders, 0.4% AI/AN, and 3.1% others (Table 2). This racial/ethnic distribution differed from that of the total PRAMS Phase 8 population. In the total PRAMS Phase 8 database, 48.3% were non-Hispanic Whites, 19.3% non-Hispanic Blacks, 20.6% Hispanic, 2.3% Asian/pacific islanders, 4.2% AI/AN, and 5.4% others.

**Table 2:** Characteristics of the study population (62,189 women for a weighted total of 3,327,782 women)

Respondent characteristics	Weighted *n*	Weighted %
Maternal age (years)		
* 20–24*	651,437	19.6%
* 25–29*	1,017,171	30.6%
* 30–34*	1,031,116	31.0%
* 35 and older*	628,058	18.8%
Race/ethnicity		
* Non-Hispanic White*	2,128,806	64.0%
* Non-Hispanic Black*	375,546	11.3%
* Hispanic*	656,786	19.7%
* Asian/Pacific Islander*	49,613	1.5%
* American Indian/Alaska Native*	13,012	0.4%
* Others*	104,020	3.1%
Marital status		
* Married*	2,190,545	65.8%
* Other*	1,136,070	34.2%
Insurance		
* Medicaid*	1,198,848	37.4%
* Private*	1,844,623	57.5%
* Self-pay*	133,171	4.2%
* Other*	29,248	0.9%
Maternal education (years)		
* Less than high school (0–11)*	333,406	10.0%
* Completed high school (12)*	756,354	23.0%
* Some college or beyond (13 or more)*	2,214,926	67.0%
Prenatal care (Kotelchuck Index)		
* Inadequate*	357,679	11.0%
* Intermediate*	313,952	9.7%
* Adequate*	1,467,448	45.1%
* Adequate Plus*	1,111,217	34.2%
Previous live birth		
* First child*	1,208,488	36.4%
* Has older sibling*	2,113,384	63.6%
Residence		
* Rural*	545,892	16.7%
* Urban*	2,729,465	83.3%
Primary language		
* English*	3,005,353	90.3%
* Other*	322,429	9.7%

The study examined barriers to accessing dental care during pregnancy, including dental coverage, primary language, provider availability, safety concerns, affordability of dental care during pregnancy, and perceived oral health benefits. The study found that women who reported that they did not have dental coverage were less likely to visit dentists for cleaning during pregnancy compared to women who had dental coverage. Moreover, women who had difficulty in finding dentists, who had safety concerns about dental care, who reported difficulty in affording dental care, and who did not perceive oral health benefits were less likely to visit dentists during pregnancy (Table 3).

**Table 3:** Bivariate analysis of the association between dental cleaning visits during pregnancy with barrier variables (62,189 women for a weighted number of 3,327,782 women)

	Dental cleaning visits during pregnancy*	*p*-value
Dental insurance		<0.001
* No*	154,166 (21.6%)	
* Yes*	1,444,858 (56.5%)	
Primary language		< 0.001
* English*	1,492,467 (49.7%)	
* Other (Spanish, Chinese)*	119,145 (37.0%)	
Difficult to find dentists who accept pregnant women		< 0.001
* No*	1,538,918 (50.4%)	
* Yes*	49,490 (27.2%)	
Difficult to find dentists who accept Medicaid		< 0.001
* No*	1,503,539 (51.1%)	
* Yes*	73,699 (26.6%)	
Safety concern		< 0.001
* No*	1,502,216 (53.6%)	
* Yes*	82,614 (18.6%)	
Difficult to afford dental care		< 0.001
* No*	1,502,208 (54.9%)	
* Yes*	82,836 (16.2%)	
Perceived oral health benefits		< 0.001
* No*	57,961 (14.7%)	
* Yes*	1,549,235 (53.2%)	

^*^Data are presented in this column as weighted number of women (*N*) and the percentage of women who had dental visits for cleaning during pregnancy (%)

After adjusting for the aforementioned confounders (age, race/ethnicity, marital status, insurance, education level, Kotelchuck, previous live birth, urban/rural, state, year), dental insurance during pregnancy and perceived oral health benefits were associated with 4.0- and 5.6-fold higher odds, respectively, of dental service utilization during pregnancy (Table 4). Lower adjusted odds of dental service utilization were associated with all other barriers; however, the relationship was not statistically significant with respect to having a primary language other than English. Therefore, primary language was not included in subsequent interaction analyses.

**Table 4:** Multivariate logistic regression analysis of the association between dental cleaning visits during pregnancy with barrier variables

Dental barrier variables	Dental cleaning visits during pregnancy	
	aOR (95% CI)	*p*-value
Dental insurance	4.03 (3.71–4.38)	< 0.001
(Ref: no dental insurance)		
Primary language is not English	0.97 (0.84–1.11)	0.615
(Ref: primary language is English)		
Difficulty finding dentists who accept pregnant women	0.62 (0.53–0.73)	< 0.001
(Ref: no difficulty to find dentists who accept pregnant women)		
Difficulty finding dentists who accept Medicaid*	0.57 (0.49–0.66)	< 0.001
(Ref: no difficulty to find dentists who accept Medicaid)		
Safety concern to go to dentist during pregnancy	0.24 (0.21–0.26)	< 0.001
(Ref: no safety concern to go to dentist during pregnancy)		
Difficulty affording dental care	0.20 (0.18–0.22)	< 0.001
(Ref. no difficulty affording dental care)		
Perceived oral health benefits	5.62 (4.97–6.35)	< 0.001
(Ref. did not perceive oral health benefits)		

After adjusting for age, marital status, insurance, education level, Kotelchuck, previous live birth, urban/rural, state, and year

^*^Among women who were enrolled in Medicaid at the time of birth

**Fig. 1:**
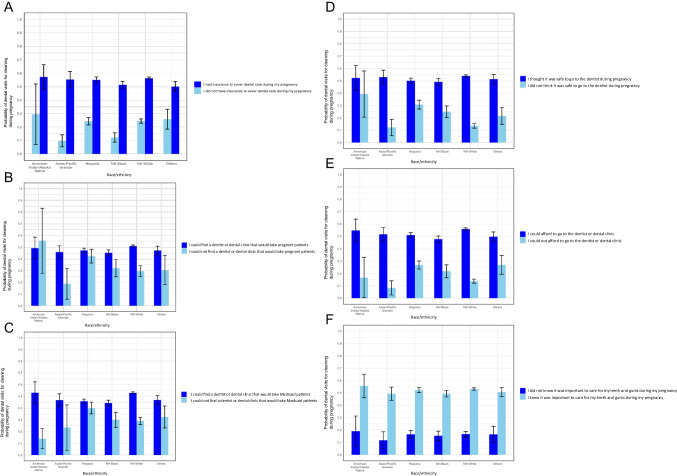
Interaction analysis between barriers to accessing dental care and probability of dental visits for cleaning during pregnancy modified by race/ethnicity. **A** Probability of dental visits for cleaning during pregnancy by dental insurance and race/ethnicity. **B** Probability of dental visits for cleaning during pregnancy by difficulty in finding dentists who accept pregnant women and race/ethnicity. **C** Probability of dental visits for cleaning during pregnancy by difficulty in finding dentists who accept Medicaid and race/ethnicity. **D** Probability of dental visits for cleaning during pregnancy by safety concerns of dental care and race/ethnicity. **E** Probability of dental visits for cleaning during pregnancy by difficulty in affording dental care and race/ethnicity. **F** Probability of dental visits for cleaning during pregnancy by perceived oral health benefits and race/ethnicity

Statistically significant effect modification by race/ethnicity was observed in crude and adjusted analyses (adjusted *p*-values reported) of the relationship between dental service utilization for all barriers included in the interaction analyses (excludes primary language) with all adjusted *p*-values < 0.001. Observed effect modification by race/ethnicity for each of the barriers is visualized in Figure 1A–F in the form of adjusted predicted probabilities and associated 95% confidence intervals of dental service utilization by race/ethnicity and the binary barrier variables. Generally, not reporting experiencing barriers yielded higher predicted probabilities of dental service utilization, regardless of race/ethnicity. Moreover, with respect to all the barriers examined, the racial/ethnic difference was attenuated for those who reported not experiencing a particular dental barrier as compared to their barrier-experiencing counterparts. The racial/ethnic difference persisted among women who did not have dental insurance during pregnancy, whereas the racial/ethnic difference was substantially attenuated among women who reported they had dental insurance. Similarly, the racial/ethnic disparity persisted among women who reported difficulty finding dentists who accepted pregnant women. The study also found that the racial/ethnic disparity persisted among women who reported safety concerns of dental care during pregnancy and women who reported they had difficulty in affording dental care during pregnancy.

## Discussion

This proposed study aimed to identify barriers to accessing dental care during pregnancy and examine how the association between these barriers to accessing dental care is modified by race/ethnicity. While the temporal relationship could not be identified from the study, the study showed that dental coverage and perceived oral health benefits during pregnancy were significantly positively associated with dental visits for cleaning during pregnancy when adjusted with other demographic variables and adequacy of prenatal care. Difficulty finding dentists who accept pregnant women, safety concerns of dental care, and the cost of dental care were also significant barriers to visiting dentists during pregnancy. Our findings related to barriers to dental service utilization during pregnancy were consistent with those of previous studies with respect to both the direction and magnitude of the observed associations [5–7, 9–18].

A multi-dimensional approach is critical from policy to practice levels to address specific barriers to visiting dentists during pregnancy. While the oral health of expecting mothers is crucial to securing and promoting the oral health of young children from a biological approach to a social-behavioral perspective, dental service utilization during pregnancy remains low, less than 50%, due to lack of dental coverage, competing priorities, or a lack of perceived benefits of oral health care during pregnancy, and a lack of willingness among dental providers to provide care due to reliability concerns. If dental coverage is the major driver to remove this barrier, primary and essential oral health services should be provided to all pregnant women by defining what those services entail. Inclusion of essential oral health care into prenatal and primary care is also aligned with the global universal health coverage momentum [19]. If difficulty finding dentists who are willing to treat pregnant women is the major barrier, policymakers need to design supporting programs and incentives for high-quality providers and facilities to serve people in marginalized communities and train dental professionals based on the national guideline to provide proper and timely dental care. If safety concerns or lack of perceived dental benefits among expecting mothers are a significant barrier to dental care during pregnancy, patient education through community-based organizations can be a reasonable solution.

The interaction analysis found that the racial/ethnic disparity in visiting dentists during pregnancy was most prominently observed among women who reported these dental barriers. In contrast, the racial/ethnic disparity was substantially attenuated among women who did not report such barriers. From this finding, we concluded that the racial/ethnic disparity could potentially be mitigated by such supporting mechanisms: dental coverage, provider availability, oral health education on the safety of dental care during pregnancy, and affordable dental care costs. Dental coverage is a critical enhancer to improve access to oral health care. Acknowledging the importance of oral health among expecting mothers, the Biden-Harris Administration announced that beginning in October 2022, all 50 states and D.C. would offer dental coverage for pregnant Medicaid enrollees [20]. Such efforts to increase dental coverage for pregnant women, especially those from underserved families, can help reduce race/ethnic oral health disparity. However, it matters what types of dental services are covered. Dental coverage is a complex system with deductibles, copays, annual cap amounts, and limitations in frequency of care delivery per patient. From the perspective of patients and providers, those services that are not covered or not covered fully may not be considered treatment options. A previous study also showed that the level of coverage mattered in dental service utilization among Medicaid-enrolled women [21].

Provider availability, measured by the subject-reported difficulty finding dentists, is another contributor to the racial/ethnic disparity in accessing and utilizing oral health care during pregnancy. While universal coverage for primary and essential oral health care for pregnant women would be critical, we must ensure dental providers are willing to examine and treat pregnant women patients following the national recommendations and guidelines. This clinical and practical guideline should be integrated into the health professional school curriculum and continuing education. Patient education is also key. Safety concern for dental care during pregnancy was among the most significant barriers to visiting dentists. The U.S. National Consensus Statement and the American College of Obstetricians and Gynecologists (ACOG) indicate that dental care is safe throughout any trimester of pregnancy [22, 23]. Providers must weigh the risks of not providing or receiving dental care over the risks of the potential harm of dental care, and both medical and dental providers should recommend timely dental care to pregnant women [24].

Subject-reported outcome research plays a significant role in bringing patients’ voices into research and policy design and understanding the target population’s needs, especially women’s perceived needs regarding oral health and access to timely dental care. Exploring and describing oral health beliefs can lead to improved care and health promotion systems specific to the target populations: racial/ethnic groups, first-time mothers, or pregnant women enrolled in Medicaid or WIC programs [14]. With mounting evidence of persisting disparities in dental service utilization during pregnancy, public and private perinatal programs and policymakers should design oral health programs that address specific barriers that pregnant women perceive, especially for women from socially disadvantaged backgrounds [25, 26]. This study highlighted significant barriers to oral health care during pregnancy, especially among racial/ethnic minority women. Future research should integrate women’s perceived oral health beliefs into the analyses to help advance the integration of oral health and women’s health during pregnancy.

## Limitations and Strengths

This secondary data analysis has a few limitations due to the nature of the survey and the retrospective design. There can be a recall bias in responding to the survey questions in addition to socially desirable responses. Dental coverage and dental visits could not be confirmed with objective data sources, such as claim data. In addition, the study may not be generalized to women who are younger than age 20 and in states other than those included in the study. It is important to acknowledge that these elements, along with vast arrays of socio-demographic factors, are multi-directionally connected beyond what was included in the study. There is a lack of temporal information to assess associations between variables. While oral health beliefs can change health behavior, pregnant women’s perceptions and attitudes toward dental care during pregnancy can change with interventions and the availability of accessible dental care [17, 27]. Future research needs to conduct a prospective study design to identify this relationship.

Despite lacking temporal information on events, the current study added valuable findings to the body of evidence regarding oral health beliefs and disparities across racial/ethnic groups of women and their dental visits during pregnancy. By examining race/ethnicity as a potential modifier of the relationships between barriers and dental service utilization, we were able to provide additional insight with respect to the transformative impact that removing these barriers might present for racial/ethnic minorities. This type of analysis is crucial for moving from a descriptive approach as it relates to the examination of racial/ethnic disparities to an inferential framework that can more rigorously identify mutable, causal targets for intervention to reduce racial/ethnic disparities in dental service utilization among pregnant women.

## Supplementary Information

Below is the link to the electronic supplementary material.

Supplementary file1 (DOCX 17 KB)

## Data Availability

The authors have the data agreement with the CDC PRAMS department. Data is available based on the agreement arrangement.

## Code Availability

Available upon request.
