# Supplementary material for: Race, Ethnicity, and Other Barriers to Access Dental Care During Pregnancy
Source: J Racial Ethn Health Disparities. 2024 Apr 26;12(3):1715–23. doi: 10.1007/s40615-024-02001-4 (PMC12069435; doi:10.1007/s40615-024-02001-4)
Supplement: Supplementary file 1 — Supplementary file1 (DOCX 17 KB) [file 40615_2024_2001_MOESM1_ESM.docx]

## Birth Certificate Variables from PRAMS

| **Category** | **Variables** | **Description** | **Categorization** |
| --- | --- | --- | --- |
| Socio-Demographic  Variables  (Intervening variables) | State | States included in PRAMS Phase 8 dataset that implemented standard oral health questions on barriers in accessing dental care during pregnancy AND met the minimum response rate for the year | 21 States  CO, CT, DC, HI, IA, IN, KY, MA, MN, MO, NC, ND, NH, NY, PR, RI, TX, UT, VA, WI, WV |
|  | Maternal age | Mother’s age at the time of birth from the birth certificate | 20-24  25-29  30-34  35 or older |
|  | Geographic location | Mother’s residence in rural or urban areas on birth certificate | Urban  Rural |
|  | Number of previous live births | from the birth certificate | First child  Previous live birth present |
|  | Maternal Education  (Number of years in school) | from the birth certificate | 0-11 (less than high school)  12 (completed high school)  13 and more (beyond high school) |
|  | Marital Status | from the birth certificate | Married  Other |
|  | Method of payment at the time of birth | from the birth certificate | Private medical insurance  Medicaid  Self-pay  Others |
| Behavior variable (Intervening variable) | Prenatal visit score (Kotelchuck Index) | from the birth certificate | Inadequate  Intermediate  Adequate  Adequate Plus |
| Race/ethnicity (Moderating variable) | Maternal Race/Ethnicity | from the birth certificate | Non-Hispanic White  Non-Hispanic Black  Hispanic  Asian/Pacific Islander  American Indian / Alaskan Native  Others |
